# Application of Particle Swarm Optimization Algorithm in the Heating System Planning Problem

**DOI:** 10.1155/2013/718345

**Published:** 2013-07-01

**Authors:** Rong-Jiang Ma, Nan-Yang Yu, Jun-Yi Hu

**Affiliations:** ^1^School of Mechanical Engineering, Southwest Jiaotong University, Chengdu 610031, China; ^2^CSR Qishuyan Institute Co., Ltd., Changzhou 213011, China

## Abstract

Based on the life cycle cost (LCC) approach, this paper presents an integral mathematical model and particle swarm optimization (PSO) algorithm for the heating system planning (HSP) problem. The proposed mathematical model minimizes the cost of heating system as the objective for a given life cycle time. For the particularity of HSP problem, the general particle swarm optimization algorithm was improved. An actual case study was calculated to check its feasibility in practical use. The results show that the improved particle swarm optimization (IPSO) algorithm can more preferably solve the HSP problem than PSO algorithm. Moreover, the results also present the potential to provide useful information when making decisions in the practical planning process. Therefore, it is believed that if this approach is applied correctly and in combination with other elements, it can become a powerful and effective optimization tool for HSP problem.

## 1. Introduction

Humanity faces serious energy and environment problems at present. The environment is increasingly threatened. For instance, with the increase of greenhouse gas emissions in the atmosphere the environments have already reached concerning levels in terms of their potential to cause climate change. Air pollution, acid precipitation, and stratospheric ozone depletion are other serious environmental concerns. The severity of climate change impacts shows the increasing trend if significant action is not taken to reduce greenhouse gas emissions [1]. An important action to address energy and environmental challenges lies in the intelligent and efficient use of energy, including reducing energy waste and using low-carbon fuels. 

In China, heating utilities have been developed rapidly, but the energy consumption of production and transport is still too much, which accounts for 21.5% of building energy consumption; building energy consumption accounts for 20.9% of social total energy consumption [2]. With the perfection of the systematic reform, the adjustment of energy structure in China and the requirement of environmental protection, heating energy structure had been changing, and it had been promoting the development of heating mode. It has very important significance to analyze, evaluate, and select heating mode correctly which suits its local characteristics. With the speeding up of urbanization, more and more heating systems will be built due to the importance of infrastructure in urban area. The research on optimal plan of heating system is very imperative for saving project investment, decreasing heating energy consumption, and improving enterprise benefit.

Sustainable development of heating system requires application of planning procedures, which includes optimization of both demand and supply sides of heating. Because the heat source site selection and heating pipe network optimizing plan have an important role in the HSP, there are many scholars concerning this subject and lots of optimization methods have been proposed. The methods of HSP can be classified into three separate categories [3]: planning by models, planning by analogy, and planning by inquiry. The planning by models can be based either on econometric or optimization models. Econometric models utilize mathematical or statistical methods and relay on statistical data. Optimization model allows for the identification of best possible solution—minimization or maximization of objective function, with the predefined set of constrains which describes the space of acceptable solutions. The planning by analogy utilizes the simulation of heating system. That kind of HSP is usually used for the verification of planning results which were achieved by other planning methods [4]. The planning by inquiry is used in the case when other aforementioned methods are not reliable. Good example of planning by inquiry is DELPHI method, which is based on the questioning of group of heating, ventilating, and air conditioning (HVAC) experts or municipal planners and statistical evaluation of their answers [5]. All the methods of HSP listed earlier have a limited transparency, especially for decision makers who do not have good mathematical background. Those methods do not give opportunity to create decision makers preference model or define that model a priori. Hence, many scholars have carried out extensive and deep research on optimization method of HSP. Shen and He [6] investigated optimal planning method of central heating system of water boiler and then put forward optimal planning model and solving method. According to the method, it can be determined the size, location, and so forth of regional heating plant and intermediate heat exchanger station, but there were no further discussions about how to design the pipe network. Wang et al. [7–9] investigated design method of central heating system with double “duct-station,” proposing two-step optimization method, but this method was only applicable to double “duct-station” system. For solving the problem, the study in [8] used fully stratified sequence method [10–12] simultaneously taking into account only one objective function for each layer of heating source layout optimization and pipe network system. However, for HSP problem, there are kinds of complex logic even iterative relationships between/among objective functions of layers. This method usually can provide the optimal solution of each layer, but it cannot ensure that the solution of the objective function for last layer is the optimal solution of the whole system exactly. Shi and Li [13] first applied genetic algorithm (GA) for solving the heating source location problem in the study. This method described the cost of the heat source and heating substation as the function of heat load and described the cost of the heating network as the function of heat load and pipe length simply. So the calculated result by this method and actual situation often put in certain error. Shi et al. [14, 15] and Mu et al. [16] put forward the relatively consistent mathematical model for heating system optimization, based on the life cycle cost method, but formula or method for some of parameters was not given clearly and integrally in the model. It seems that limitation is inevitable in the process of the practical application of these methods. But we noticed the life cycle cost (LCC) and particle swarm optimization (PSO) algorithm in the more extensive research areas.

Life cycle cost (LCC) has been applied since the 1960s when the United States' Department of Defense stimulated the development and application of LCC to enhance its cost effectiveness. Defense systems, such as an aircraft or a special land vehicle, are ideal for LCC analyses since the Department of Defense mainly controls the entire life cycle [17]. LCC may be defined as “the cost of acquisition, ownership, and disposal of a product over a defined period of its life cycle” [18, 19]. LCC is a standard engineering economic approach used for choosing among alternative products or designs that approximately provide the same service to the customer [20]. In many cases it may not be necessary to perform a complete LCC analysis, but rather to estimate the differences between the alternatives for the major cost elements [21]. The LCC process may also provide information, for example, in the assessment of the economic viability of products and projects, in the identification of the cost drivers and cost efficiency improvements, and in evaluations of different strategies for product operation, maintenance, and inspection, and so on [22].

There are two popular swarm inspired methods in computational intelligence areas: ant colony optimization (ACO) and particle swarm optimization (PSO). ACO was inspired by the behaviors of ants and has many successful applications in discrete optimization problems. The particle swarm concept originated as a simulation of simplified social system. The original intent was to graphically simulate the choreography of a bird block or fish school. However, it was found that particle swarm model can be used as an optimizer. A substantial review of the properties of the global optimization problems has been given by Parsopoulos and Vrahatis [23]. As one of the global optimization problems, PSO has been widely used in various kinds of planning problems, especially in the area of substation locating and sizing [24–27]. But in area of heating supply, PSO is mainly applied in heating load forecasting [28, 29], but rarely used in HSP.

The main objective of this paper is to discuss the usefulness of the PSO algorithm for solving the HSP problem. Therefore, based on the LCC approach, an integral mathematical model is presented and PSO algorithm is introduced and improved for solving the problem. In the end, the results of the case study suggest the effectiveness of improved particle swarm optimization (IPSO) application to the optimal planning method for heating system.

## 2. Mathematical Formulation

### 2.1. Problem Definition and Assumptions

LCC is related to the systems engineering process, because economic considerations are very important in the process of creating systems. Life cycle economic analyses should be done early in the system or product life cycle, because the outcome of the systems engineering process cannot be influenced very much when the design is completed. Thus, LCC involves evaluation of all future costs related to all of the phases in the system life cycle including design, construction and/or production, distribution, operation, maintenance and support, retirement, and material disposal, and so on [30].

Cost models may range from simple to complex and are essentially predictive in nature. Parameters, such as the system's physical environment, usage demand, reliability, maintainability, labor, energy, taxes, inflation, and the time value of money, may have a great influence on the life cycle costs [17].

The main objective of this paper is to discuss the usefulness of the PSO algorithm for owners in making sustainable heating system investment decisions and to improve their decision-bases for municipal administration. Therefore, we apply LCC approach to describe the HSP problem.

Moreover, HSP considered in this study works under the following definition and assumptions.

- A heat consuming installation can connect with any heat source but cannot connect with two or more heat sources at the same time.
- The indirect connection between heat consuming installation and heat source is not allowed.
- A heat source must be connected with more than one heat consuming installation; otherwise, it will be closed.
- Any connection between any two heat sources is not allowed.
- The location of heat consuming installation is fixed.
- A heat source can be sited in a given region.
- The elevation difference between heat consuming installation and heat source is ignored.
- Heating system planning and optimization can be achieved by changing the number and the heating capacity of heat source and the distance between the heat source and heat consuming installation.
- The measure between heat source and heat consuming installation is simplified to the Manhattan (or city block) distance.
- There is no functional difference between any two heat sources and their products.

### 2.2. Notation

The notations used in the mathematical formulations are given as follows.

*Indices*

*i*: Optional heating source
*k*: Heating equipment
*j*: Heat consuming installation
*r*: Heat load distributing segment.

*Parameters*

 
*m*: Number of heat source
 
*k*_*i*_: Number of heating equipment which could be installed at the heating source *i*; *k*_*i*_ = {1,2, …, *P*_*i*_}
 
*n*: Number of heat consuming installation
 
*n*_*r*_: Number of heat load distributing segments
 
*F*_*i*_: Life cycle fixed cost of the heat source *i*
 
*F*_*ik*_: Life cycle fixed cost of the heating equipment *k* which is in the heat source *i*
 
*C*_*ikjr*_: Variable production and transport discounted costs within life cycle of heating equipment *k* to satisfy the heat load distributing segment *r* of heat consuming installation *j*, which is in the heat source *i*; *C*_*ikjr*_ = *P*_*ikjr*_ + *t*_*ikjr*_, where *P*_*ikjr*_ is the variable production discounted cost within life cycle of specific heat load; *t*_*ikjr*_ is the transport discounted cost within life cycle per specific heat load
 
*X*_*ikjr*_: Continuous variable, the load of the heat load distributing segment *r* of heat consuming installation *j*, which is supplied by heating equipment *k* of the heat source *i*
 
*Q*_*jr*_: Load of the heat load distributing segment *r* of heat consuming installation *j*
 
*S*_*ik*_: Maximum supply capacity of heating equipment *k* of the heat source *i*
 
*Q*_*i*_^max⁡^: Maximum supply capacity of heat source *i*
 
*C*_*zd*_: Major repair depreciation discounted costs within life cycle of heat source *i*
 
*C*_*rg*_: Labor discounted cost within life cycle of heat source *i*
 
*u*: Coefficient of sum; *u* = [(1+*r*)^*y*^ − 1]/*r*(1+*r*)^*y*^, where *r* is the standard discount rate, and *y* is the life cycle
 
*P*_*rl*_: Price of fuel
 
*Q*_*w*_: Calorific value of fuel
 
*η*: Thermal efficiency of heat source
 
*E*: Water and electricity consumption costs of specific heat load
 
*h*_*r*_: Duration of heat load distributing segment *r*
 
*β*: Sulfur content in fuel
 
*λ*: Standard emission charge for SO_2_
 
*t*_*rw*_(*j*): Pipe network discounted cost per specific heat load, which is supplied by heat source *i* to heat consuming installation *j*
 
*C*(*L*_*j*_): The discounted cost of pipe segment *L*_*j*_
 
*C*_*zd*_(*L*_*j*_): The major repair depreciation discounted costs within life cycle of pipe segment *L*_*j*_
 
*C*_*sr*_(*L*_*j*_): The heat loss discounted costs within life cycle of pipe segment *L*_*j*_
 
*Q*(*L*_*j*_): The heat load-bearing of pipe segment *L*_*j*_
 
*C*_*dl*_(*L*_*j*_): The power consumption discounted cost within life cycle of pipe segment *L*_*j*_ per specific heat load
 
*t*_*rz*_(*j*): The transport discounted cost within life cycle per specific heat load, which is supplied to heat consuming installation *j*
 
*C*_*zd*_(*j*): The major repair depreciation discounted costs within life cycle of heat consuming installation *j*
 
*Q*_*ij*_: The heat load of pipe network for heat consuming installation *j*
 
*C*_*dl*_(*j*): The power consumption discounted cost within life cycle of heat consuming installation *j* per specific heat load
 
*C*_*rg*_(*j*): The labor discounted cost within life cycle of heat consuming installation *j*
 
*a*[*d*(*L*_*j*_)]^*b*^: Investment of *d*(*L*_*j*_) meters diameter double-pipe per meter length, where *a* and *b* are coefficients of pipe laying
 
*γ*: Rate of major repair depreciation
 
*ρ*: Rate of gross fixed capital formation
 
*ω*: Conversion coefficient of the units
 
*R*: Specific frictional resistance
 
*l*_*dl*_(*L*_*j*_): Equivalent length of local resistance for pipe segment *L*_*j*_
 
*H*_*gl*_: Heating period
 
*P*_*d*_: Electricity price for industrial uses
 
*η*_*xb*_: Efficiency of circulating water pump
 
*t*_*g*_, *t*_*h*_: Supply/return water temperature of pipe segment
 
*ξ*: Conversion coefficient of the units
 
*k*: Heat transfer coefficient
 
*ε*: Local heat loss coefficient of pipe fittings
 
*P*_*sr*_: Annual costs of heat loss
 
*t*_*g*,*pj*_: Annual mean supply water temperature of pipe segment
 
*t*_*h*,*pj*_: Annual mean return water temperature of pipe segment
 
*t*_*hj*,*pj*_: Annual mean temperature
 
*c*_1_, *c*_2_: Comprehensive coefficient of investment
 
*Q*_*ij*_: Heat load of pipe network
 
*α*: Correction factor
 
*μ*: Conversion coefficient of the units
 Δ*P*_*j*_: Pressure difference between supply and return water of pipe network for heat consuming installation *j*
 
*S*_*gz*_: Average annual wages of operating personnel and manager
 
*n*_*yg*_: Number of operating personnel and manager per 1 MW heat load
 
*Ω*: Conversion coefficient of the units.

*Decision Variables*

 
*Y*_*ik*_: 1, if the equipment *k* is installed or set up in the heat source *i*; 0, if the equipment *k* is not installed or set up in the heat source *i*
 
*Z*_*i*_: 1, if the heat source *i* is set up; 0, if the heat source *i* is not set up.

### 2.3. Mathematical Model of HSP

In this study, the problem is summarized into a multisource, multifacility, single-commodity, multiraw material plant location problem, and a mixed 0-1 integer planning model has been formulated. The cost model of the heat source and the heat-transmission network concerned in the optimization model are considered in this study. The objective function of heating system planning problem is to minimize the total heat production cost. The proposed mathematical model formulation for HSP problem can be found as follows.

Minimize

(1) $$\begin{array}{c} \text{LCC}=\sum_{i=1}^{m}F_{i}Z_{i}+\sum_{i=1}^{m}\;\sum_{k=1}^{k_{i}}F_{ik}Y_{ik}+\sum_{i=1}^{m}\;\sum_{k=1}^{k_{i}}\;\sum_{j=1}^{n}\;\sum_{r=1}^{n_{r}}C_{ikjr}X_{ikjr} \end{array}$$

subject to

(2) $$\begin{array}{c} \sum_{i=1}^{m}\;\sum_{k=1}^{k_{i}}X_{ikjr}=Q_{jr},\;j=1,2,\ldots ,n;\;\;r=1,2,\ldots ,n_{r}, \end{array}$$

(3) $$\begin{array}{c} \sum_{j=1}^{n}\;\sum_{r=1}^{n_{r}}X_{ikjr}\leq S_{ik}Y_{ik},\;i=1,2,\ldots ,m;\;\;k=1,2,\ldots ,k_{i}, \end{array}$$

(4) $$\begin{array}{c} \sum_{k=1}^{k_{i}}S_{ik}Y_{ik}\leq Q_{i}^{\max},\;i=1,2,\ldots ,m, \end{array}$$

(5) $$\begin{array}{c} \sum_{k=1}^{k_{i}}Y_{ik}\leq P_{i}Z_{i},\;i=1,2,\ldots ,m, \end{array}$$

(6) $$\begin{array}{c} Z_{i},Y_{ik}=0\;\text{or}\;1,\;\;X_{ikjr}\geq 0. \end{array}$$

Objective function (1) minimizes the discounted costs within life cycle of heating system as the general objective; it is an index of dynamic economy evaluation, where *F*_*i*_, *F*_*ik*_, and *C*_*ikjr*_ are composed of respective discounted costs together. Constraint (2) is each heat consuming installation's heat load, which is heat consumption for each user and the requirements of the heating quantity and quality. Constraint (3) means that each of the heating equipment in the heating system bear heat load should not exceed the maximum heating capacity. Constraint (4) means the maximum heating capacity of heating source, which is allowed under the restrictions of objective conditions. Constraint (5) means that only open heating source first can install equipment in it. In the model, there are two decision variables, in which *Z*_*i*_ is related to heating source, and *Y*_*ik*_ is related to heating equipment. 

Because the piecewise function of heat load duration curve is introduced in the process of solving the model, this model can be applied to any form of heating system. 

### 2.4. Formulation of Heating System Cost Model

#### 2.4.1. The Heating Source Cost Model

The heating source cost model is aimed to resolve the calculation problem of *F*_*ik*_, in the objective function (1), and *P*_*ikjr*_, which is a part of *C*_*ikjr*_ in the objective function (1). It consists of fixed costs and variable costs, the former refers to all necessary costs of heating source, so long as open a heating source or install a piece of heating equipment, the latter only associated with the size of the heat load and running status of equipment. Consider

(7) $$\begin{array}{c} F_{ik}=C_{zd}+C_{rg}, \end{array}$$

(8) $$\begin{array}{c} P_{ikjr}=u\left(\frac{0.36P_{rl}+0.72\beta \lambda }{Q_{w}\eta }+0.36E\right)h_{r}. \end{array}$$

Equation (7) is the formulation of heating source fixed costs, and (8) is the formulation of heating source variable costs.

#### 2.4.2. The Heating Network Cost Model

The heating network cost model is aimed to resolve the calculation problem of *t*_*ikjr*_, which is a part of *C*_*ikjr*_ in the objective function (1), and also to optimize the direction of heating network and the pipe diameter. Heating network (heat consuming installation included) cost consists of the heating network operation cost and heat consuming installation costs. Heating network operation cost consists of major repair depreciation discounted cost, power consumption discounted cost, pipe network heat loss discounted cost, and labor discounted cost. By dividing the discounted cost within life cycle of pipe segment allocation to the total heat load bearded by itself directly and evenly, the transport discounted cost within life cycle per specific heat load can be obtained, which is supplied by heat source *i* to heat consuming installation *j*. Consider

(9) $$\begin{array}{c} t_{ikjr}=t_{rw}\left(j\right)+t_{rz}\left(j\right), \end{array}$$

(10) $$\begin{array}{c} t_{rw}\left(j\right)=t_{rw}\left(j-1\right)+C\left(L_{j}\right),\\ C\left(L_{j}\right)=\frac{C_{zd}\left(L_{j}\right)+C_{sr}\left(L_{j}\right)}{Q\left(L_{j}\right)}+C_{dl}\left(L_{j}\right),\\ t_{rw}\left(0\right)=0, \end{array}$$

(11) $$\begin{array}{c} C_{tz}\left(L_{j}\right)=a{\left[d\left(L_{j}\right)\right]}^{b}l\left(L_{j}\right), \end{array}$$

(12) $$\begin{array}{c} C_{zd}\left(L_{j}\right)=\gamma \rho C_{tz}\left(L_{j}\right)u, \end{array}$$

(13) $$\begin{array}{c} C_{dl}\left(L_{j}\right)=\frac{2\omega R\left[l\left(L_{j}\right)+l_{dl}\left(L_{j}\right)\right]uH_{gl}P_{d}}{\eta_{xb}\left(t_{g}-t_{h}\right)}, \end{array}$$

(14) Csr(Lj)=ξkπd(Lj)l(Lj)(1+ε) ×HglPsru(tg,pj+th,pj−2thj,pj).

Equation (9) is the heating network transportation cost model. The pipe segment cost model is composed of (10)–(14), where (10) is the pipe network discounted cost per specific heat load supplied by heat source *i* to heat consuming installation *j*; (11) is the investment cost of pipe segment *L*_*j*_; (12) is the major repair depreciation discounted cost of pipe segment *L*_*j*_; (13) is the power consumption discounted cost of pipe segment *L*_*j*_ per specific heat load; and (14) is the heat loss discounted cost of pipe segment *L*_*j*_. Consider

(15) $$\begin{array}{c} t_{rz}\left(j\right)=\frac{C_{zd}\left(j\right)}{Q_{ij}}+C_{dl}\left(j\right)+C_{rg}\left(j\right), \end{array}$$

(16) $$\begin{array}{c} C_{tz}\left(j\right)=c_{1}+\alpha c_{2}Q_{ij}, \end{array}$$

(17) $$\begin{array}{c} C_{zd}\left(j\right)=\gamma \rho C_{tz}\left(j\right)u, \end{array}$$

(18) $$\begin{array}{c} C_{dl}\left(j\right)=\frac{\mu \Delta P_{j}}{\eta_{xb}\left(t_{g}-t_{h}\right)}H_{gl}P_{d}u, \end{array}$$

(19) $$\begin{array}{c} C_{rg}\left(j\right)=S_{gz}n_{yg}u\Omega . \end{array}$$

The heat consuming installation cost model is composed between (15) and (19), where (15) is the transport discounted cost within life cycle per specific heat load supplied by heat consuming installation *j*; (16) is the investment cost of heat consuming installation *j*; (17) is the major repair depreciation discounted cost of heat consuming installation *j*; (18) is the power consumption discounted cost of heat consuming installation *j* per specific heat load; and (19) is the labor discounted cost of heat consuming installation *j*.

## 3. PSO and Its Improvement

### 3.1. PSO Algorithm

The PSO is proposed by Kennedy and Eberhart [31, 32] in 1995, and the motivation for the development of this algorithm was studied based on the simulation of simplified animal social behaviors, such as fish schooling and bird flocking. Similar to other population-based optimization methods such as genetic algorithms, the particle swarm algorithm starts with the random initialization of a population of particles in the search space [33]. However, unlike in other evolutionary optimization methods, in PSO there is no direct recombination of genetic material between individuals during the search. The PSO algorithm works on the social behavior of particles in the swarm. Therefore, it provides the global best solution by simply adjusting the trajectory of each individual toward its own best location and toward the best particle of the entire swarm at each time step (generation) [31, 34, 35]. The PSO method is becoming very popular due to its simplicity of implementation and ability to quickly converge to a reasonably good solution.

### 3.2. Formulation of General PSO

Specifically, PSO algorithm maintains a population of particles, each of which represents a potential solution to an optimization problem. The position of the particle denotes a feasible, if not the best, solution to the problem. The optimum progress is required to move the particle position in order to improve the value of objective function. The convergence condition always requires setting up the move iteration number of particle.

The position of particle move rule is shown as follows:

(20) $$\begin{array}{c} V_{s}\left(t+1\right)=wV_{s}\left(t\right)+C_{1}r_{1}\left(P_{s}-X_{s}\left(t\right)\right)+C_{2}r_{2}\left(G-X_{s}\left(t\right)\right), \end{array}$$

(21) $$\begin{array}{c} X_{s}\left(t+1\right)=X_{s}\left(t\right)+V_{s}\left(t+1\right), \end{array}$$

where *V*_*s*_(*t*) represents the velocity vector of particle *s* in *t* time; *X*_*s*_(*t*) represents the position vector of particle *s* in *t* time; *P*_*s*_ is the personal best position of particle *s*; *G* is the best position of the particle found at present; *w* represents inertia weight; *C*_1_, *C*_2_ are two acceleration constants, called cognitive and social parameters, respectively; and *r*_1_ and *r*_2_ are two random functions in the range [0,1]. 

**Figure 1:**
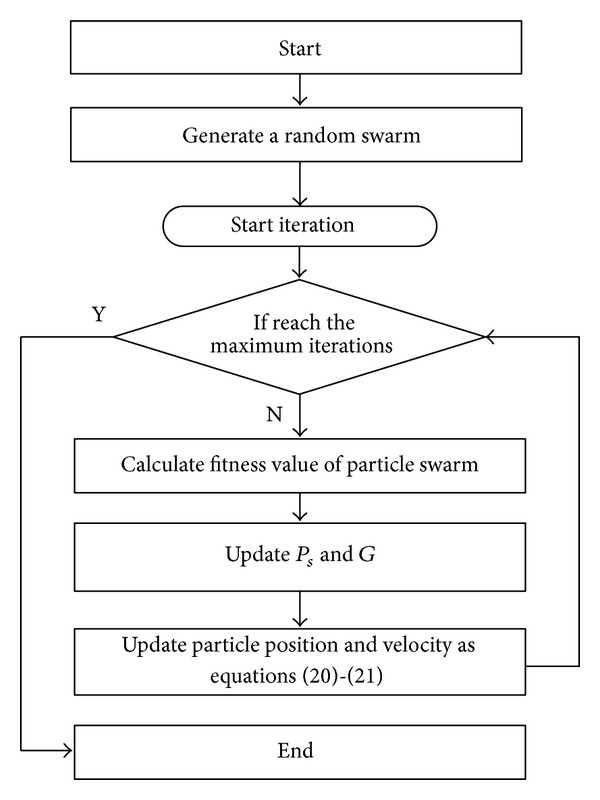
Flow chart of general PSO.

The flow chart of general PSO is shown in Figure 1.

### 3.3. Improvement of Particle Swarm Optimization (IPSO) for HSP Problem

For HSP problem and its model in this paper, the value of LCC depends mostly on the distance between heating source and heat consuming installation, and the number of heating source *i*. It is necessary to make corresponding improvements on PSO, in order to solve this problem more accurately and effectively.

The evolution of the solution set begins with an initial solution set in the PSO; initial solution set is composed of initial particles. Each solution location is represented by an *i*-dimensional vector; *i* represents the number of variables of each solution, and it represents the number of heating sources in particularly in this paper.

The position coordinate of heating source (*p*) has two components, which is represented by two *i*-dimensional vectors, where *x* direction coordinates are represented by vector *px*, and *y* direction coordinates are represented by vector *py*. Therefore, *x* direction component for the position vector of particle *s* in *t* time can be represented by *px*_*s*_(*t*), and the rest can be done in the same manner.

In the same way, the velocity for location change of heating source (*Vp*) has two components, which is represented by two *i*-dimensional vectors, where *x* direction component for the velocity vector is represented by vector *V* *px*, and *y* direction component for the velocity vector is represented by vector *V* *py*. Therefore, *x* direction component for the velocity vector of particle *s* in *t* time can be represented by *V* *px*_*s*_(*t*), and the rest can be done in the same manner.

Thus, the update rule of velocity for each particle is indicated by (22)-(23), and the update rule of position for each particle is indicated by (24)-(25). Consider

(22) Vpxs(t+1)=wVpxs(t)+C1r1(Ps−pxs(t)) +C2r2(G−pxs(t)),

(23) Vpys(t+1)=wVpys(t)+C1r1(Ps−pys(t)) +C2r2(G−pys(t)),

(24) $$\begin{array}{c} px_{s}\left(t+1\right)=px_{s}\left(t\right)+Vpx_{s}\left(t+1\right), \end{array}$$

(25) $$\begin{array}{c} py_{s}\left(t+1\right)=py_{s}\left(t\right)+Vpy_{s}\left(t+1\right). \end{array}$$

The meanings of parameters are consistent with previous description.

### 3.4. Calculated Flow of**  **IPSO

The calculated flow of proposed IPSO is described as follows. 

#### 3.4.1. Initial Solution

The initial solution for HSP problem is obtained by random initial position of each heat source; a matrix is employed in recording the coordinates and the heat load-bearing information of heat source, and the calculated flow of initial solution is as follows.

1. Set up the number of heat source *i*, and generate an empty matrix for the initial position of heat source.
2. Based on randomly and evenly distributed manner, generate the position coordinates of heat sources, into the matrix.
3. Call the decoding function; calculate the heat load-bearing and the cost for each heat source, into the matrix.
4. Calculate the LCC, the fitness value of the initial particle.

#### 3.4.2. Decoding Function

In this paper, decoding function will call the matrix for current position and heat load of heat consuming installation, and then according to the matrix for the position of heat source, which is represented by current particle, divide the heating range of each heat source, and calculate the LCC.

Information matrix of heat consuming installation (*heat_point*) is a *j*-line four-column matrix; the first column represents the serial number of heat consuming installation, the second column represents the *x* coordinate of heat consuming installation, the third column represents the *y* coordinate of heat consuming installation, and the heat load of heat consuming installation is represented by the fourth column. The calculated flow of initial solution is as follows.

1. Read matrix *heat_point*, and let *j* = *j* + 1.
2. Calculate the distance to all heat source from the heat consuming installation *j*, into the vector *l*_*j*_.
3. By substituting *l*_*j*_ into (10)–(14), calculate the cost of the heat consuming installation *j* connected with each heat source.
4. Find out the minimum cost, and the heat consuming installation *j* connected with the corresponding heat source.
5. If *j* is the last heat consuming installation then stop; otherwise, go to Step 1.

#### 3.4.3. The Evolution of Particle Swarm

After one generation of particles, a new generation is evolved as follows.

1. Call the decoding function; calculate the fitness value of the particle swarm.
2. Update the individual optimal solution *P*_*s*_ and the global optimal solution *G*.
3. Update the speed vector, by using (22)-(23).
4. Update the speed vector, by using (24)-(25).

#### 3.4.4. Improvement Approach

The PSO's convergence is fast, so it is liable to fall into local optimal solution. In order to improve the optimizing capability, we add modular arithmetic of velocity vector into each iterative operation. If the norm of velocity vector *V* is less than the predetermined minimum value *V*_min⁡_, then generate a random velocity, let the current particle swarm out of local convergence region, and search other solution spaces. However, after it falls into local optimal solution, the norm of velocity tends to 0 in the general PSO algorithm, the solution stabilized near the local optimal solution, and it cannot explore search space furthermore. 

**Figure 2:**
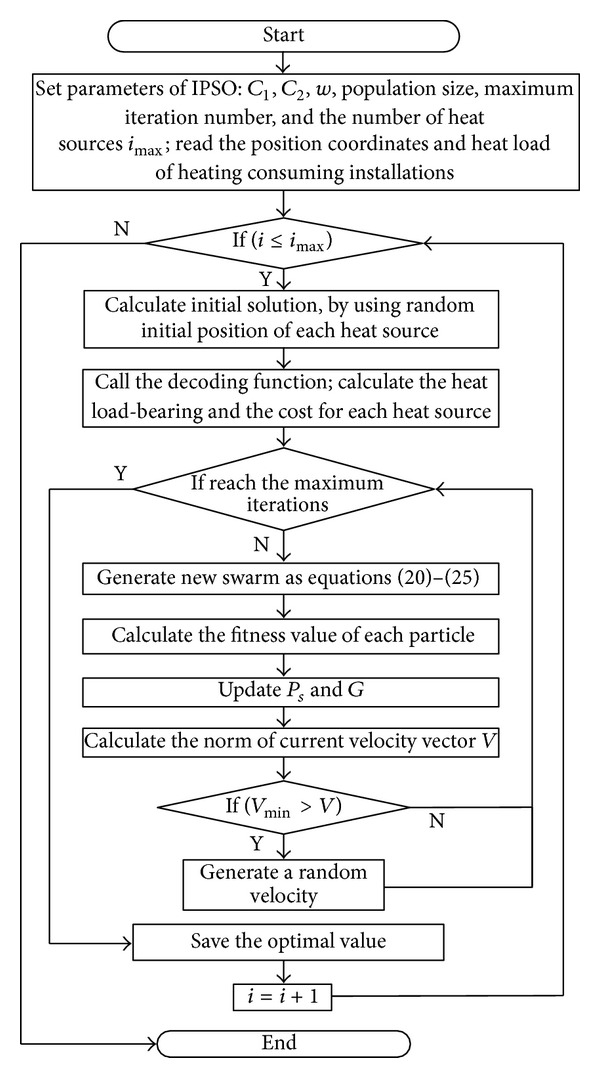
Flow chart of IPSO.

The flow chart of IPSO is shown as Figure 2.

## 4. Case Study

### 4.1. Basic Information of Case

**Figure 3:**
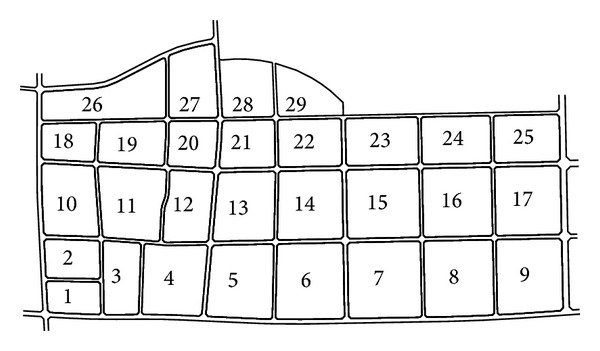
Site location plan of 29 heating districts.

**Figure 4:**
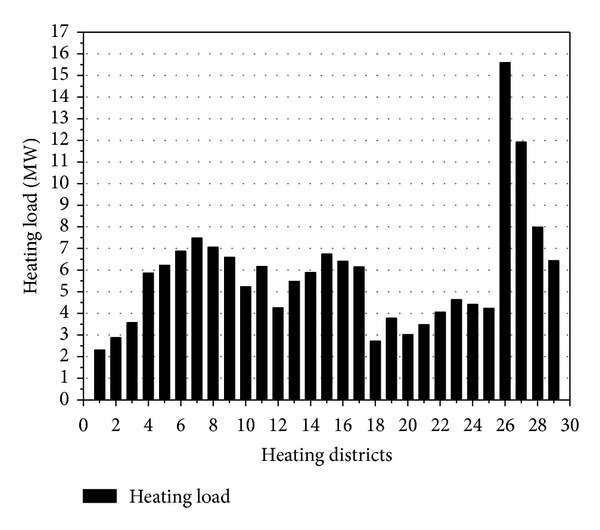
Heating load of 29 heating districts.

This is a heating plan for a new area in China covering the area of 3.346 million square meters, and heat load is 167.3 MW in total. Based on the road network, the new area is divided into 29 heating districts (Figure 3), and the heating load of each district (Figure 4) is supplied by their small gas-fired boiler.

### 4.2. The Parameters of Algorithms

The role of the inertia weight *w*, in (20), (22), and (23), is considered critical for the PSO's convergence behaviour. The inertia weight is employed to control the impact of the previous history of velocities on the current one. Accordingly, the parameter *w* regulates the trade-off between the global and local exploration abilities of the swarm. A large inertia weight facilitates global exploration, while a small one tends to facilitate local exploration. A suitable value for the inertia weight *w* usually provides balance between global and local exploration abilities resulting in a reduction of the number of iterations required to locate the optimum solution. Initially, the inertia weight was constant. However, experimental results indicated that it is better to initially set the inertia to a large value, in order to promote global exploration of the search space, and gradually decrease it to get more refined solutions [32, 36]. Thus, an initial value around 1.2 and a gradual decline towards 0 can be considered as a good choice for *w*. The parameters *C*_1_ and *C*_2_, in (20), (22), and (23), are not critical for PSO's convergence. However, proper fine-tuning may result in faster convergence and alleviation of local minima. A further study of the acceleration parameter in the first version of PSO is given in [37]. As default values, *C*_1_ = *C*_2_ = 2 were proposed, but experimental results indicate that *C*_1_ = *C*_2_ = 0.5 might provide even better results. Some work reports that it might be even better to choose a larger cognitive parameter, *C*_1_, than a social parameter, *C*_2_, and *C*_1_ + *C*_2_ ≤ 4 [38, 39], but (*C*_1_ + *C*_2_)/2 = 1.494 was suggested by [35]; the strategy of acceleration parameter linear changing with iterations was proposed by Ratnaweera et al. [40], but acceleration parameter is the nonlinear function of the ratio *G*-to-*P*_*s*_, which was proposed by Arumugam et al. [41]; Jie et al. [42] suggested to adjust the acceleration coefficient by measuring diversity.

But so far, the research on the most appropriate values for *w*, *C*_1_, and *C*_2_ has no unified conclusion. And how the variable values impact the solution to HSP problem is unknown. For HSP problem on kinds of values is unknown. So we set the *w*, *C*_1_, and *C*_2_ to common values in this study.

### 4.3. Analysis of Results

By applying PSO and IPSO algorithm, respectively, we solved the HSP problem in this paper. The parameters of PSO and IPSO are summarized in Table 1.

**Figure 5:**
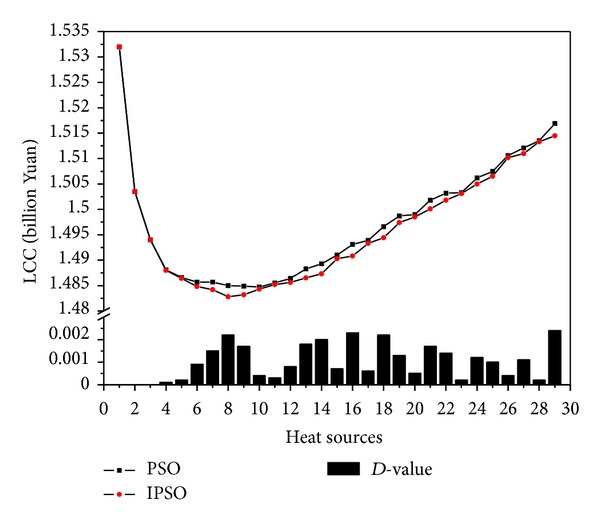
Algorithm calculation results comparison (optimum value).

**Figure 6:**
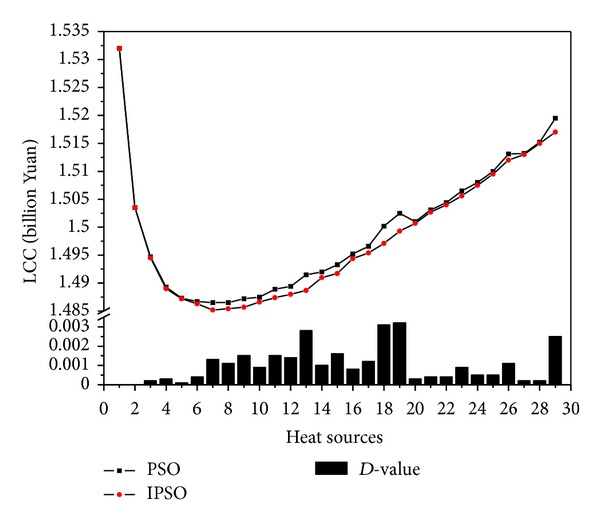
Algorithm calculation results comparison (average value).

In this study, 29 kinds of schemes of heating (from one heat source to twenty-nine heat sources) were calculated for 10 times through reading initial conditions from the excel file successively, which contains the coordinates and heat load of heat consuming installation, preset maximum number of heat source. The results of LCC and the *D*-value for the optimum and the average between PSO and IPSO at the same number of heat source are shown as Figures 5 and 6 and Table 2.

From analyzing the results, we can draw the following conclusion about the HSP problem.

1. The original plan (the heating load of each district is supplied by its small gas-fired boiler) is not an economic and reasonable plan for the case, and the LCC is the second highest in 29 schemes, which is only better than the scheme which plans to set up one heat source only.
2. From one heat source to twenty-nine heat sources, LCC is monotone decreasing until a minimum value first, then monotone increasing.
3. Only one minimum value of LCC that appeared throughout the change process, which is 1.4828 billion Yuan, the scheme of which plans to set up 8 heat sources, is the best choice for the case. (The detailed calculation results of this scheme are shown in Table 3.)

By observing the algorithms, the following is also concluded.

1. The optimal solution of IPSO is better than PSO. The optimum LCC which calculated by IPSO is not larger than PSO for all 29 schemes. The maximum *D*-value is 2.4 million Yuan in the scheme which plans to set up 29 heat sources.
2. The real minimum LCC was not calculated by PSO. The minimum LCC calculated by PSO is 1.9 million Yuan larger than the minimum LCC calculated by IPSO.

**Figure 7:**
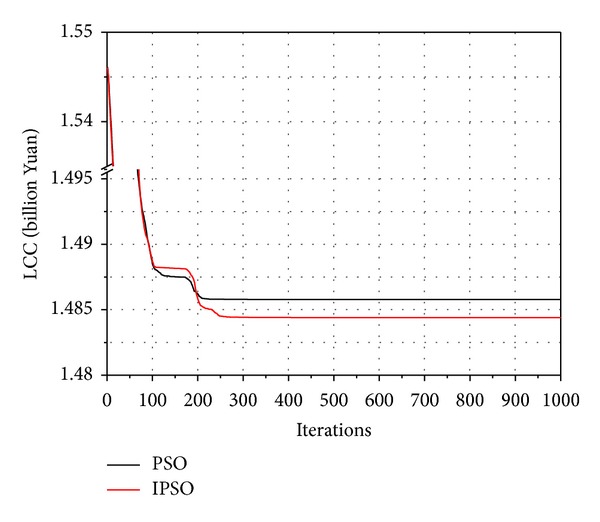
Algorithm calculation comparison (7 heat sources).

Figure 7 compares the LCC convergence curves of two algorithms in three kinds of schemes, respectively. When the population size and the iteration number of PSO are same as those of IPSO during the HSP optimization process, although the PSO algorithm is faster for giving the optimization results, but the optimal results by IPSO are better than the searcher values by PSO. The main reason for current performance is that IPSO can avoid local optimal solution and then further expand the search space so as to find a better solution. 

Hence, it can be concluded that the improvement approach is effective, and the proposed method IPSO has better significance in solving the HSP problem and competitive to PSO algorithm.

## 5. Discussion

**Figure 8:**
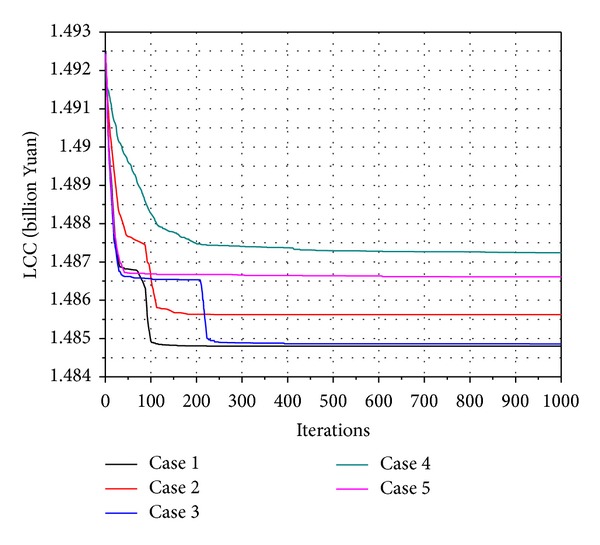
Algorithm calculation in comparison with different parameters (6 heat sources).

**Figure 9:**
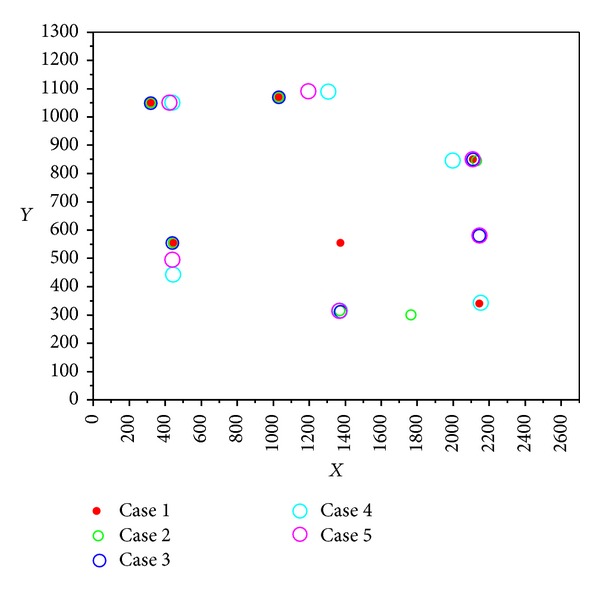
Coordinates of heating sources in comparison with different parameters (6 heat sources).

Section 4.2 referred to the values of *w*, *C*_1_, and *C*_2_ which may influence the computational results. In Figures 8 and 9, the results obtained by IPSO were also proved. Algorithm calculation comparison with different parameters is shown in Figures 8 and 9, which is a visual display of the coordinates of heating sources with different parameters.

The parameters of each case in the figure are summarized in Table 4.

The influence aspect of the algorithm is worth further study, but because of the major goal of the present study, the more details were not presented here and will be discussed in a separate paper. 

## 6. Conclusions and Prospects

1. In this paper, we presented an integral mathematical model for solving the heating system planning (HSP) problem taking into account minimizing the cost of heating system for a given life cycle time.
2. According to the particularity of HSP problem, the particle swarm optimization (PSO) algorithm was introduced and improved, the new definition and update rule of velocity and position vector were proposed, and the improvement approach about generating a random velocity was adopted to avoid particle swarm into local optimal solution. Then an actual case study was calculated to check its feasibility in practical use. The results show that the IPSO algorithm can more preferably solve the HSP problem than PSO algorithm.
3. Although there is no more discussion about the influence of computational results by changing the values of algorithm parameters (*w*, *C*_1_, and *C*_2_), but the results of the case study still show the potential to provide useful information when making decisions in the practical planning process. Thus, it is believed that if this approach is applied correctly and in combination with other elements, such as the accurate prediction of heating load, the running efficiency of equipment, and the real operation situation, it can become a powerful and effective optimization tool for HSP problem.

## Figures and Tables

**Table 1:** PSO and IPSO parameters.

Variable	Symbol	Value	
		PSO	IPSO
Population size	—	100	100
Maximum iteration number	—	1000	1000
Inertia weight	*w*	0.7	0.7
Acceleration constant	*C*_1_	2	2
	*C*_2_	2	2

**Table 2:** Algorithm calculation results comparison.

Heat source	LCC (billion Yuan)					
	Optimum value			Average value		
	PSO	IPSO	*D*-value	PSO	IPSO	*D*-value
1	1.5320	1.5320	0.0000	1.5320	1.5320	0.0000
2	1.5035	1.5035	0.0000	1.5035	1.5035	0.0000
3	1.4940	1.4940	0.0000	1.4947	1.4945	0.0002
4	1.4881	1.4880	0.0001	1.4893	1.4890	0.0003
5	1.4866	1.4864	0.0002	1.4873	1.4872	0.0001
6	1.4857	1.4848	0.0009	1.4867	1.4863	0.0004
7	1.4857	1.4842	0.0015	1.4865	1.4852	0.0013
8	1.4850	1.4828	0.0022	1.4865	1.4854	0.0011
9	1.4849	1.4832	0.0017	1.4872	1.4857	0.0015
10	1.4847	1.4843	0.0004	1.4875	1.4866	0.0009
11	1.4855	1.4852	0.0003	1.4889	1.4874	0.0015
12	1.4864	1.4856	0.0008	1.4894	1.4880	0.0014
13	1.4883	1.4865	0.0018	1.4915	1.4887	0.0028
14	1.4893	1.4873	0.0020	1.4920	1.4910	0.0010
15	1.4910	1.4903	0.0007	1.4933	1.4917	0.0016
16	1.4931	1.4908	0.0023	1.4952	1.4944	0.0008
17	1.4939	1.4933	0.0006	1.4966	1.4954	0.0012
18	1.4966	1.4944	0.0022	1.5002	1.4971	0.0031
19	1.4987	1.4974	0.0013	1.5025	1.4993	0.0032
20	1.4990	1.4985	0.0005	1.5010	1.5007	0.0003
21	1.5018	1.5001	0.0017	1.5031	1.5027	0.0004
22	1.5032	1.5018	0.0014	1.5044	1.5040	0.0004
23	1.5033	1.5031	0.0002	1.5065	1.5056	0.0009
24	1.5062	1.5050	0.0012	1.5080	1.5075	0.0005
25	1.5075	1.5065	0.0010	1.5100	1.5095	0.0005
26	1.5106	1.5102	0.0004	1.5131	1.5120	0.0011
27	1.5121	1.5110	0.0011	1.5132	1.5130	0.0002
28	1.5135	1.5133	0.0002	1.5152	1.5150	0.0002
29	1.5169	1.5145	0.0024	1.5195	1.5170	0.0025

**Table 3:** The detailed results of 8 heat sources scheme.

Heating source	Coordinate	Supply heat load (MW)	Heat consuming installation
1	(395, 555)	24.38	1, 2, 3, 10, 11, 12
2	(320, 1050)	22.07	18, 19, 26
3	(1030, 315)	24.43	4, 5, 6, 13
4	(760, 1070)	14.92	20, 27
5	(1745, 555)	24.73	7, 14, 15, 23
6	(1040, 1090)	21.94	21, 22, 28, 29
7	(2380, 850)	14.78	17, 24, 25
8	(2145, 340)	20.05	8, 9, 16

**Table 4:** The parameters of each case in Figures 8 and 9.

Variable	Symbol	Value				
		Case 1	Case 2	Case 3	Case 4	Case 5
Population size	—	100	100	100	100	100
Maximum iteration number	—	1000	1000	1000	1000	1000
Inertia weight	*w*	0.7	0.4	0.9	0.7	0.7
Acceleration constant	*C*_1_	2	2	2	0.2	3.8
	*C*_2_	2	2	2	0.2	3.8
